# The effect of psychosocial and healthcare interventions on reducing hospitalization in people with dementia: an umbrella review

**DOI:** 10.1002/alz.71323

**Published:** 2026-07-20

**Authors:** Connie Howard, Alba Fernández‐Sanlés, Nawal Abukar, Daniel Davis, Claire Goodman, Sanjiv Gupta, Melanie Handley, Naaheed Mukadam, Carol Riddington, Kate Walters, Gill Livingston, Andrew Sommerlad

**Affiliations:** ^1^ Division of Psychiatry University College London London UK; ^2^ Institute of Health Informatics University College London London UK; ^3^ Centre for Research in Public Health & Community Care (CRIPACC) University of Hertfordshire Hatfield UK; ^4^ North London NHS Foundation Trust London UK; ^5^ Centre for Ageing Population Studies University College London London UK; ^6^ Research Department Primary Care & Population Health University College London London UK

**Keywords:** dementia, emergency department, healthcare use, hospitalization, psychosocial interventions

## Abstract

People with dementia are at increased risk of hospitalization compared to individuals without dementia, yet the efficacy of psychosocial and healthcare interventions in reducing hospitalization is unclear. We conducted an umbrella review examining the effects of psychosocial and healthcare interventions compared to controls on reducing hospitalization in people with dementia. We included 25 systematic reviews comprising 77 unique studies totaling 1,483,077 participants. There was high‐certainty evidence that case management and exercise programs had no effect on reducing hospitalization, low‐certainty evidence that advance care planning reduced hospital admissions, and moderate‐certainty evidence that clinical pharmacists in multidisciplinary teams (MDTs) reduced medication‐related hospital re‐admissions. The evidence for psychosocial and healthcare interventions in reducing hospitalization in dementia is insufficient to make strong positive recommendations, but there are indications that advance care planning and clinical pharmacists in MDTs may be beneficial. High‐quality randomized controlled trials are required to establish more confident clinical recommendations.

## Highlights

- Umbrella review of 25 reviews comprising 77 unique papers and 1,483,077 participants.
- Interventions previously thought to be effective, such as case management programs and encouraging exercise, have no effect on hospitalization rates or length of stay.
- There is moderate certainty evidence that including clinical pharmacists in multi‐disciplinary teams reduces medication‐related hospital admissions of people with dementia.
- There is low‐certainty evidence that advance care planning reduces hospitalization.
- There is a lack of evidence for other interventions to accurately gauge their effectiveness.

## 1 INTRODUCTION

The number of people living with dementia (PwD) globally is expected to increase from 57 million in 2019 to 152 million by 2050.^1^ As PwD have a 42% increased risk of admission to acute hospital care compared with those without dementia, even after adjusting for physical comorbidities, there will be substantially increased hospital use.^2^ In the United Kingdom (UK), PwD occupy one in six hospital beds, with £2.8 billion annual costs due to hospital inpatient admissions, and it is expected that by 2040, one quarter of hospital inpatients will have dementia.^3^ Once admitted, PwD stay in acute hospitals up to seven times longer than age‐matched patients^4^ and are at a 7% to 35% increased risk of readmission.^5^, ^6^ Admissions to acute hospitals can also have a significant negative impact on the health of PwD, including increased mortality^7^, ^8^, ^9^, ^10^ and frailty,^9^, ^11^ more inpatient complications,^12^, ^13^ and further cognitive and functional decline^8^, ^9^, ^13^, ^14^, ^15^, ^16^ with only 42% of PwD recovering to their pre‐hospitalization level of function.^17^ Acute hospitals are fast‐paced environments that are usually not specifically designed to care for PwD,^9^, ^18^, ^19^ with staff who often report feeling inadequately prepared and supported to look after PwD.^3^, ^20^

While some hospital admissions are essential and appropriate, 20% to 49% of hospitalizations of PwD are potentially preventable,^4^, ^21^, ^22^ often as defined as admissions for ambulatory care‐sensitive conditions (ACSCs). ACSCs are health concerns such as urinary tract infections or pain that could theoretically have been treated on an outpatient basis through early intervention, preventing admission,^23^ and these admission rates are 78% higher in people with dementia.^24^ It is therefore crucial to consider how to reduce these admissions. Psychosocial and healthcare interventions may affect hospitalization risk by improving the person with dementia's or their family carer's ability to manage self‐care and medication regimens, developing coping mechanisms and the ability to seek appropriate early health‐seeking behaviors, improving neuropsychiatric symptoms, or other mechanisms. However, current evidence examining the effectiveness of psychosocial and healthcare interventions on reducing hospitalization in PwD is spread across different intervention types, with unclear efficacy.^25^ Given the breadth and heterogeneity of interventions and outcome measures in this field, an updated systematic review of primary studies would risk inappropriate statistical pooling and limited comparability. We therefore chose an umbrella review,^26^ to assess the consistency, strength, and gaps in the evidence base across intervention domains by synthesizing findings from existing systematic reviews and meta‐analyses.

Therefore, our aim was to conduct an umbrella review to synthesize the evidence on the efficacy and quality of psychosocial and healthcare interventions aimed at reducing hospitalization rates in PwD.

## 2 METHODS

We registered our umbrella review in PROSPERO and report our umbrella review according to Preferred Reporting Items for Overviews of Reviews (PRIOR) guidelines^27^ (Table S1). We systematically searched the literature for published systematic reviews and meta‐analyses that examined the association between psychosocial and healthcare interventions and hospitalization (used as an overarching term encompassing hospital admissions and readmissions, length of stay [LoS] or delayed discharge, and emergency department [ED] visits) in PwD.

### 2.1 Information sources/search strategy

We searched systematically across four electronic databases from inception – MEDLINE, Embase, Cochrane, and CINAHL – in November 2024, with no language or date restrictions. Searches were re‐run on September 15, 2025, to ensure recent reviews were included. Keywords and Medical Subject Headings terms such as “hospitalization,” “emergency room visits,” and “dementia” were combined with Boolean operators to identify relevant systematic reviews and meta‐analyses. See Table S2 for the complete search strategy.

### 2.2 Selection process

Duplicates were removed using Covidence software. Two reviewers (C.H. and N.A.) independently screened titles and abstracts, followed by full‐text articles according to the predefined eligibility criteria, with detailed reasons for exclusion given in Table S3. At each stage, disagreements were discussed with G.L. and A.S. to reach a consensus. Inter‐rater reliability was assessed using Cohen's kappa, with values >0.81 considered almost perfect agreement, 0.61 to 0.80 substantial, 0.41 to 0.60 fair, and <0.40 no/slight agreement.^28^

### 2.3 Eligibility criteria

Eligibility criteria were established using the Population, Intervention, Comparator, Outcome, Study type (PICOS) framework. Systematic reviews were eligible for inclusion if they included (1) PwD (population), (2) any psychological/social support/healthcare delivery interventions (intervention), (3) a suitable control group (control), and (4) a measure of hospitalization (outcome) and (5) were systematic (study type), defined as studies seeking to “collate all empirical evidence that fits pre‐specific eligibility criteria to answer a specific research question” using explicit, predefined methods to minimize bias.^29^ Eligible study designs included randomized controlled trials (RCTs), non‐randomized studies of interventions (NRSIs), observational studies with intervention analysis, quasi‐experimental studies, and mixed‐method studies.

Systematic reviews were excluded if they focused on mild cognitive impairment (MCI) or delirium or if they analyzed the effects of pharmacological interventions on hospitalization. Studies were not eligible if they did not examine an intervention compared to a control. Therefore, qualitative studies, cross‐sectional studies, editorials, letters, and abstracts were also excluded.^26^

### 2.4 Data extraction process

We created a custom document to extract the following data: author, publication year, location, methods (total relevant studies, number of RCTs, and observational studies), population (total relevant sample size, inclusion and exclusion criteria, mean age, and dementia severity information), intervention (intervention type, control group, setting, target, and length), results for each outcome, and quality assessments. One reviewer (C.H.) independently extracted data from each review according to our custom template, and a second reviewer (N.A.) checked for any discrepancies. To visualize overlap in primary studies between reviews, two reviewers (C.H. and N.A.) created a citation matrix (Figure S1), which was structured according to the interventions used in each primary study.

Where a primary study intervention was categorized differently between reviews, one reviewer (C.H.) examined the primary study to determine the study description and consulted with N.A., A.F.‐S., G.L., and A.S. to determine the accurate intervention label and adjusted the matrix accordingly (changes presented in Figure S1). Similarly, where individual study sample sizes were reported differently across reviews, one reviewer (C.H.) assessed the primary study to determine its true sample size. These verified sample sizes were then used to calculate the umbrella review's total sample size. The corresponding authors of systematic reviews were contacted^30^, ^31^ for clarification if they used unpublished data that did not correspond to the primary study.

### 2.5 Risk of bias assessment

Risk of bias was assessed using A Measurement Tool to Assess Systematic Reviews (AMSTAR II tool),^32^ which was designed to assess RCTs and NRSIs. Critical domains in the assessment include the pre‐registration of review methods, comprehensive search strategies, justification of excluded studies, a satisfactory analysis of the risk of bias and publication bias, and the use of appropriate statistical combination methods. One reviewer (C.H.) independently rated all reviews according to these criteria and consulted A.S. and G.L. when unsure. See Table S4 for full AMSTAR II ratings.

### 2.6 Synthesis methods

We present our findings in a narrative synthesis. Due to study heterogeneity and a subsequent lack of meta‐analyses in many systematic reviews, we could not conduct meta‐analyses.

### 2.7 Certainty assessment

The Grading of Recommendations, Assessment, Development, and Evaluation (GRADE) tool^33^ was used to assess the certainty of evidence. One reviewer (C.H.) assessed all included systematic reviews utilizing this framework, with a second reviewer (A.F.‐S.) independently assessing a random 10% in full to check for consistency. After this, the research team (C.H., A.F.‐S., G.L., and A.S.) discussed the GRADE ratings of all interventions and resolved any uncertainty by coming to a consensus and through rechecking data if necessary.

According to this approach, all observational studies began as low‐quality evidence, and RCTs began as high‐quality evidence.^34^ Five criteria concern the downgrading of evidence: risk of bias, inconsistency, indirectness, imprecision, and publication bias, and three criteria gave the opportunity to upgrade the evidence: magnitude of effect, dose–response gradient, and plausible confounders. As some reviews included multiple interventions, with different effect estimates per intervention, the GRADE tool was applied to each intervention in each review so the ratings could make recommendations by intervention type.^35^ According to these criteria, each intervention within each review was then categorized as having either “high,” “moderate,” “low,” or “critically low” certainty of evidence. When a study was reported across multiple reviews with different GRADE ratings, we included the study in both categories. We had a priori decided to prioritize higher‐quality review evidence over lower‐quality evidence in our synthesis of the evidence. Full GRADE ratings are provided in Table S5.

### 2.8 Public and patient involvement (PPI)

PPI members with experience caring for family members with dementia were involved in the umbrella review from its inception. Quarterly meetings were held with five members to discuss the review and its progress. PPI members assisted in creating a comprehensive search strategy, introducing lived experience to the interpretation of findings and identifying crucial areas for future research. Two members (C.R., S.G.) were involved as co‐authors of the article.

## 3 RESULTS

**FIGURE 1:**
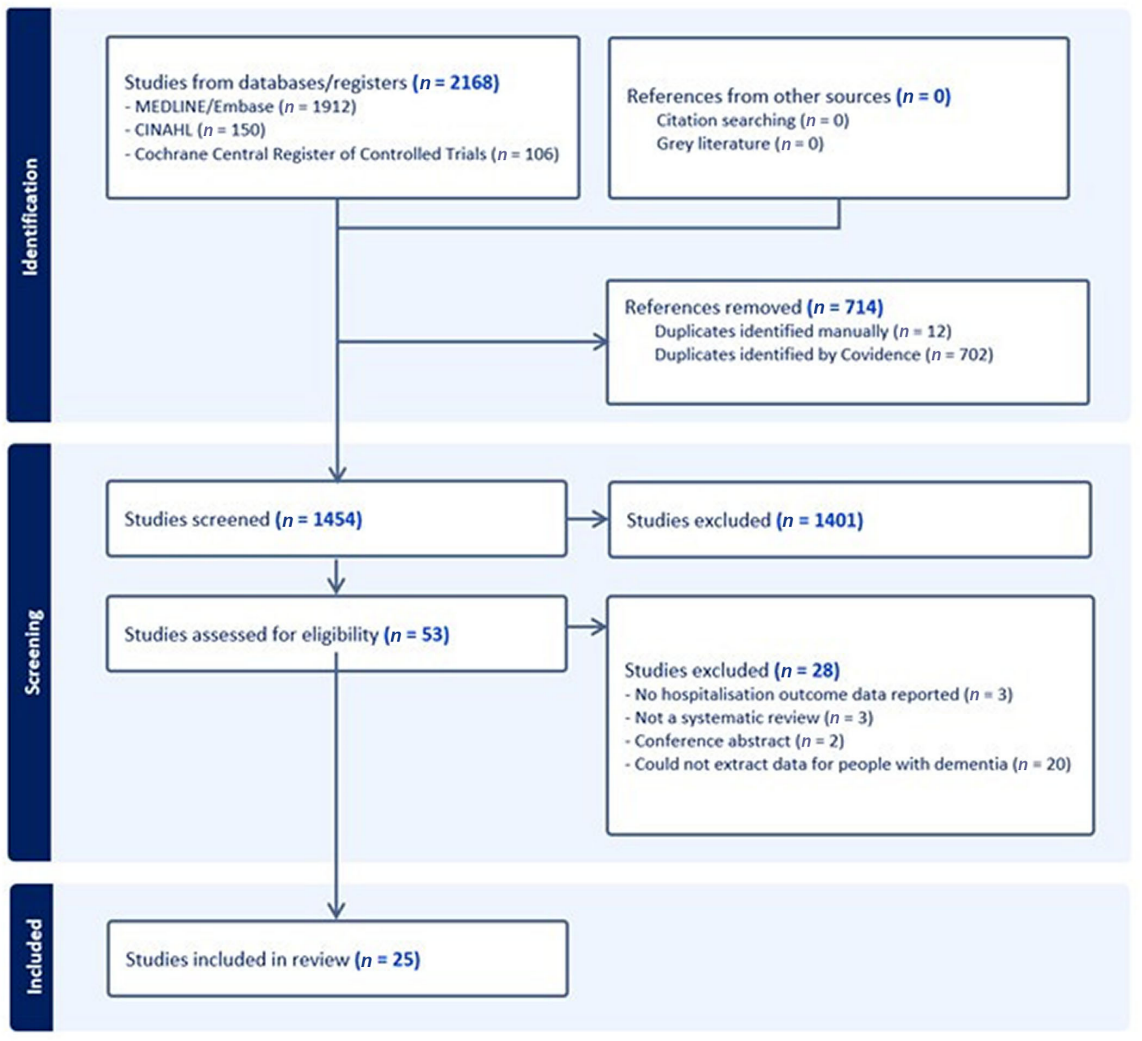
Preferred reporting items for systematic reviews and meta‐analyses (PRISMA) global education and career consultants diagram.

The initial search yielded 1828 studies, with 136 additional articles found in September 2025 (see Figure 1 for flow of studies). After deduplication, 1454 studies were eligible for screening. Following title and abstract screenings (*n* = 1454) and full text review (*n* = 53), 25 reviews were selected for data extraction. Inter‐rater agreement was substantial at the title and abstract screening stage (κ = 0.72) and at full‐text screening (κ = 0.80).

Seventy‐seven unique studies with 1,483,077 participants, of whom 977,946 came from a single scoping review (RCTs = 47, NRSIs = 30) were included. Twenty‐two studies (28.6%) appeared in multiple reviews (Figure S1 for citation matrix showing overlap). Most studies were conducted in Europe (*n* = 38), followed by North America (*n* = 29), Australia (*n* = 5), East Asia (*n* = 4), and Southeast Asia (*n* = 1).

Twelve of 25 reviews (48%) assessed multiple intervention types. There was a range of intervention types, and we grouped interventions into broad categories to allow for efficient synthesis of findings. Most of the reviews examined the effects of case management (*n* = 15) on hospitalization of PwD, followed by multidisciplinary team (MDT) management (*n* = 9), physical activity interventions (*n* = 4), education/training programs (*n* = 4), type of hospital setting (*n* = 3), group activities (*n* = 3), post‐operative/discharge interventions (*n* = 2), palliative care (*n* = 2), counseling (*n* = 2), volunteering (*n* = 2), carer interventions (*n* = 1), and advance care planning (*n* = 1). Studies and the definitions of interventions they included are summarized in Table 1. According to AMSTAR II criteria (Table S4), three reviews were high quality,^19^, ^30^, ^36^ eight were moderate quality,^31^, ^37^, ^38^, ^39^, ^40^, ^41^, ^42^, ^43^ four were low quality,^25^, ^44^, ^45^, ^46^ and 10 were critically low quality.^5^, ^47^, ^48^, ^49^, ^50^, ^51^, ^52^, ^53^, ^54^, ^55^ Issues leading to risk of bias and downgrading of quality mainly related to study protocols not being pre‐specified, search strategies that were insufficiently comprehensive, and inadequate methods for assessing risk of bias.

**TABLE 1:** Descriptive table of systematic reviews including the types of studies reviewed, setting and outcome.

Review ID	Location	No. relevant studies (no. of RCTs)	No. relevant participants	Intervention type	Control group	Intervention setting	Intervention target	Outcome measured
Case management								
Pimouguet (2010)^47^	North America	3 (3)	659	Interaction between case manager and patient–caregiver dyads providing support, information, continuity, and advocacy for community services, financial and legal matters, and disease evolution.	Usual care	Community	PwD or PwD–carer dyad	Hospital admission LoS ED visit
Tam‐Tham (2012)^31^	North America	3 (3)	509	At least one healthcare professional including at least one component of assessment and planning, education, emotional support, service facilitation, or legal/financial advice	Usual care	Community	PwD or PwD–carer dyad	Hospital admission ED visit
Frost (2020)^49^	North America and Southeast Asia	3 (1)	3665	Post‐diagnostic care from a primary care provider, including but not limited to structured needs assessments, caregiver support, referrals, and psychoeducation	Usual care	Community	PwD or PwD–carer dyad	Hospital admission Readmission LoS ED visit
Phelan (2015)^50^	North America and Europe	8 (8)	2321	Case management/care coordination and multidisciplinary assessment and management (including exercise, safety, and carer support groups)	Not specific	Community	PwD–carer dyad	Hospital admission LoS ED visits
Quinn (2020)^37^	North America	1 (1)	780	Telephone‐based collaborative dementia care providing education, support, and care coordination	Usual care	Community	PwD	Hospital admission ED visits
Khanassov (2014)^46^	East Asia	2 (2)	180	Structured needs assessments, care plans, education and support programs, and finance skills	Usual care	Community	PwD or PwD– carer dyad	Hospital admission
Lee (2020)^25^	North America and Europe	8 (7)	2666	Care coordination providing support, counseling information, follow‐up meetings, telehealth education programs, etc.	Usual care	Community	PwD or PwD–carer dyad	Hospital admission
Reilly (2016)^30^	North America and Europe	5 (5)	905	Any intervention in the community focused on planning and coordinating the needs of the PwD	Usual care	Community	PwD or PwD–carer dyad	Hospital admission LoS ED visits
Demanes (2022)^52^	North America	3 (3)	1411	Care coordinators providing telephone‐based education, linkage to services, skill building, informal counseling	Usual care	Community	PwD or PwD–carer dyads	Hospital admission ED visit
Hovsepian (2022)^39^	North America	12 (9)	9472	Home/community‐based care models prioritizing needs and developing care plans.	Usual care	Community	PwD or PwD–carer dyad	Hospital admission LoS
Ma (2019)^5^	North America	2 (0)	1300	Family‐centered function‐centered care or collaborative dementia care	Usual care	Acute care	PwD or PwD–carer dyad	Hospital admission Readmission LoS
Casarez (2024)^41^	North America	1 (0)	84	Family‐focused function‐centered care to maximize function and physical activity	Usual care	Acute care	PwD and PwD‐ carer dyad	LoS
Packer (2019)^53^	North America and Europe	8 (8)	1943	Collaborative care coordination, including education, caregiver coping skills, legal and financial advice, goal‐oriented support group, reassessments	Usual care	Community	PwD and PwD–carer dyad	Hospital admission LoS
Godard‐Sebillotte (2018)^45^	North America and Europe and East Asia	11 (11)	2936	EPOC taxonomy: “delivery arrangements,” “financial arrangements,” “governance arrangements,” or “implementation strategies”	Usual care	Community	PwD or PwD–carer dyad	Hospital admission LoS ED visit
Sawan (2021)^51^	North America	1 (0)	1216	Discharge planning, follow‐ups, comprehensive assessment of carer stress and patients' behavioral and psychological condition, medication review, caregiver counseling, and reviewing medical regimen	Usual care	Community	PwD and PwD–carer dyad	Hospital admission
Multidisciplinary teams (MDT)								
Tunnard (2022)^40^	North America and Europe	3 (1)	106+	Collaborative virtual (scheduled and unscheduled) MDT meetings to discuss care management options	Standard care	Care home	Staff caring for PwD	Hospital admission
Ma (2019)^5^	Europe	1 (1)	460	A pharmacist is included in the care team, completing medication reconciliations, medication reviews, and participating in ward rounds	Usual care	Acute care	PwD	Readmission
Feast (2020)^19^	Europe	1 (0)	101	Multidisciplinary postoperative program to reduce postoperative complications	Usual care	Acute care	PwD	LoS
Packer (2019)^53^	North America	1 (1)	100	Systematic multidisciplinary assessments in assisted living residences	Usual care	Care home	PwD	Hospital admission
Godard‐Sebillotte (2019)^45^	Europe	1 (1)	256	Comprehensive geriatric assessment by a geriatrician or old‐age psychiatrist before care home placement	Usual care	Community	PwD	ED visits
Phelan (2015)^50^	North America	1 (0)	100	Multidisciplinary assessments during relocation to assisted living	Usual care	Care home	PwD	Hospital admission ED visits
Andrews (2025)^42^	Europe	1 (1)	429	Pharmacist‐led medication reconciliation and medication review	Usual care	Acute care	PwD	Hospital admissions ED visits
Lee (2025)^43^	North America	1 (0)	365	Multicomponent recommendations by ER^2^ tool. Recommendations include (1) medication review by ED physician; (2) discharge planning team; (3) occupational therapy; (4) reorienting patient during temporal disorientation	Usual care	Acute care	PwD	Hospital admissions
Smith (2025)^55^	North America	1 (0)	977,946	Post‐acute home healthcare including skilled nursing, therapy, social work, and home healthcare services	Skilled nursing facilities	Community	PwD	Readmission
Physical activity								
De Souto Barreto (2020)^93^	Europe	6 (6)	1312	Group‐based supervised multicomponent exercise training programs	Non‐exercise group	Long‐term care or community	PwD	Hospital admission
Phelan (2015)^50^	Europe	1 (1)	135	Home‐based occupational therapy with a caregiver for individuals at a memory clinic/day clinic in a geriatric department	Not specified	Community	PwD or PwD– carer dyad	Hospital admission
Lee (2020)^25^	Europe	1 (1)	210	Supervised moderate‐intensity exercise, including aerobic, strength training, and balance exercises	Usual care	Community	PwD or PwD–carer dyad	Hospital admission
Packer (2019)^53^	Europe	3 (3)	446	Physiotherapy and occupational therapy in home‐based and day care settings	Usual care	Community	PwD	Hospital admission LoS
Education/Training								
Godard‐Sebillotte (2019)^45^	Europe	1 (1)	390	Self‐management with educational material and meetings	Usual care	Community	PwD or PwD–carer dyad	Hospital admissions LoS
Casarez (2024)^41^	Europe and Australia	2 (0)	729	Training program to improve dementia care practices or an integrated approach to support staff and promote confidence	Before and after implementation	Acute care	PwD or staff caring for PwD	LoS
Andrews (2025)^42^	Australia and East Asia	2 (0)	206	Education of MDT and pharmacist developed individualized deprescribing protocols, or education materials sent to general practitioners with anticholinergic medications tapered or replaced	Before and after implementation or acute care	Community	Staff caring for PwD	Hospital admission ED visits
Bocks (2025)^54^	Australia	1 (1)	1304	IMPETUS‐D online training program with modules covering best practice palliative and end‐of‐life care, recognizing deterioration due to dementia versus delirium and recognizing dying	Usual training	Community	Staff caring for PwD	Hospital admissions
Type of hospital setting								
Reich (2022)^44^	Europe	2 (0)	200	Single rooms versus multi‐bed wards	Before implementation	Acute care	PwD	LoS Discharge
McCausland (2018)^38^	Europe	2 (1)	1500	Specialist inpatient dementia units	Standard inpatient care	Acute care	PwD	Readmission LoS Discharge
Feast (2020)^19^	Europe	1 (0)	48	Specialist cognitive geriatric unit	Conventional geriatric care	Acute care	PwD	LoS
Lee (2025)^43^	Europe	1 (0)	801	Geriatric emergency medicine unit for elderly patients	Usual care	Acute care	PwD	Readmissions
Group activities								
Feast (2020)^19^	Europe	1 (0)	85	10‐week music program	Usual care	Acute care	PwD	LoS
Reich (2022)^44^	Europe	1 (0)	85	10‐week music program	Before implementation	Acute care	PwD	LoS
Packer (2019)^53^	Europe	2 (2)	213	Day‐care center three times a week offering social, physical, and occupational activities	Usual care	Community	PwD–carer dyad	Hospital admission
Discharge								
Sawan (2021)^51^	Europe and East Asia	2 (0)	433	MDT administered intervention, including caregiver education and counseling, tailored medication management advice, and discharge planning	Usual care	Acute care or community	PwD or PwD–carer dyad	Readmission LoS
Ma (2019)^5^	Europe	1 (0)	390	Post‐discharge telephone follow‐up	Usual care	Acute care	PwD	Readmission
Palliative care								
Quinn (2020)^37^	Australia	1 (1)	64	Palliative Care Planning Coordinator performing Facilitated case conferencing at nursing homes	Usual care	Care home	PwD	Hospital admission ED visits
Ma (2019)^5^	North America	1 (0)	368	Home‐ and clinic‐based palliative care provided by trained specialist palliative care teams. The program consists of in‐home medical consultation, ongoing evidence‐based prognostication, caregiver support, and advanced healthcare planning	Usual care	Community	PwD	Readmission
Counseling								
Packer (2019)^53^	Europe	2 (2)	666	Individual and group‐based counseling sessions plus educational courses or reminiscence therapy for 12 weeks with rotating weekly topics	Usual care	Community	PwD or PwD–carer dyad	Hospital admission ED visits
Godard‐Sebilotte (2019)^45^	North America and Europe	2 (2)	552	Individual and group‐based counseling sessions plus educational courses	Usual care	Community	PwD or PwD‐ carer dyad	Hospital admission LoS
Volunteering								
Reich (2022)^44^	Europe	1 (0)	16	Take part in activities with school‐aged volunteers 1 day per week for 2 h	Before implementation	Acute care	PwD	Readmission
Feast (2020)^19^	Australia	1 (0)	548	Person‐centered volunteer program to help with eating/drinking, interacting with others, engaging in therapeutic activities	Usual care	Acute care	PwD	Readmission
Carer interventions								
Godard‐Sebillotte (2019)^45^	North America	1 (1)	197	REACH II caregiver intervention providing education, social support, cognitive strategies for reframing negative emotional responses and strategies for enhancing healthy behaviors	Usual care	Community	Carers	Hospital admission ED visits
Advance care planning (ACP)								
Dixon (2018)^48^	North America and Europe	6 (4)	479,231	ACP staff training program or ACP discussion/facilitation or written advance directives	Before and after implementation	Care home or community	PwD or PwD–carer dyad	Hospital admission LoS ED visit

Abbreviations: ED, emergency department; LoS, length of stay; PwD, people with dementia.

The GRADE rating for each intervention within each review (Table S5) showed high certainty of evidence for findings from two reviews on case management and physical activity. All 11 primary studies in these reviews were RCTs, with 905 participants receiving case management and 1312 participants in the exercise program. Seven reviews provided moderate certainty of evidence, and these evaluated case management, palliative care, MDTs, and type of hospital setting interventions in 24 primary studies (21 RCTs) with a pooled sample of 10,904 participants. Twelve reviews had low certainty of evidence and evaluated case management, physical activity, MDTs, advance care planning (ACP), education/training, group activities, counseling, and carer interventions in 40 primary studies (33 RCTs) with 495,490 participants. Eight reviews had critically low certainty and evaluated case management, palliative care, type of hospital setting, MDTs, education/training, discharge, group activities, and volunteering in 19 primary studies (one RCT) with 975,980 participants. Common reasons for downgrading evidence GRADE were issues with risk of bias and imprecision.

### 3.1 Case management

Based on previous definitions,^46^, ^56^, ^57^ case management was categorized as an intervention with a clinical lead (case manager) responsible for advocating for, planning, and facilitating healthcare services to support the individual and family member care needs to promote high‐quality outcomes; remote/virtual delivery of case management was assessed in some studies. Results are summarized in Table 2.

**TABLE 2:** Summary of evidence and quality of findings for effect of case management interventions on hospitalization in people with dementia.

Quality	No. studies (RCTs), sample size	Intervention subtype	Results
High	5 (5) 905	Case management	Hospital admissions No effect^30^ (OR 0.87 [0.59,1.3]) LoS Increased Meta‐analysis of three studies^30^: (MD 0.63 [0.40, 0.86])^59^, ^60^, ^61^ Reduced in hospital units or residential homes in case management:^58^ − After 6 months (MD −5.8 [−7.93, −3.67]) − After 12 months (MD −7.7 [−9.38, −6.02])
Moderate	15 (13) 7544	Case management	Hospital admissions No effect Meta‐analysis of three studies: (RR 1.00 [0.76, 1.33])^31^ Individual studies: (RR 1.12 [0.87, 1.68]),^63^ (RR 1.20 [0.64, 2.25]),^60^ (RR 0.93 [0.69, 1.24]),^71^ (RR 1.37 [0.38, 4.94]),^66^ (RR 1.16 [0.67, 1.99]),^59^ (RR 0.57 [0.27, 1.22]),^58^ (RR 0.88 [0.35, 2.21]),^67^ (RR 1.21 [0.87, 1.68]),^69^ (RR 1.04 [0.93, 1.17]),^70^ (MD −0.08 [−0.29, 0.13]),^65^ (RR 1.86 [0.84, 4.09]),^72^ (DID −1 [−13, 11])^62^ Reduced (RR 0.46 [0.23, 0.92]) – unpublished data^74^ LoS No effect (MD 0.03 [−0.25, 0.27]),^63^ (MD −0.16 [−0.39, 0.07]),^64^ (MD −0.15 [−0.51, 0.20]),^59^ (MD −2.10 [−6.9, 2.70]),^58^ (MD −1.5 [−6.14, 3.14]),^67^ (MD 0.16 [−1.69, 1.91]),^69^ (MD 0.06 [−0.10, 0.22])^70^ Reduced (MD −0.37 [−0.66, −0.08]),^68^ (MD −0.34 [−0.57, 0.12]) – unpublished data^74^ Increased (MD 3.80 [3.07, 4.53])^72^ ED visits No effect (RR 0.73 [0.32, 1.63]),^58^ (RR 0.80 [0.35, 1.84]),^67^ (RR 1.35 [0.85, 2.15]),^68^ (RR 1.06 [0.78, 1.44]),^69^ (RR 0.84 [0.49, 1.44]),^72^ (MD −0.17 [−0.56, 0.22]),^65^ (DID −1 [−15, 12])^62^ Reduced (0.23 ± 0.59 vs 0.09 ± 0.32)^73^
	2 (2) 938	Virtual case management	Hospital admissions No effect (*β* 0.09 [−0.05, 0.2]) at 6 months,^80^ (*β* −0.03 [−0.18, 0.12]) at 12 months^75^ ED visits Reduced (*β* −0.14 [−0.29, −0.01]) at 12 months^75^
Low	16 (13) 7027	Case management	Hospital admissions No effect Individual studies^60^, ^62^, ^63^, ^64^, ^65^, ^66^, ^69^, ^76^: Meta‐analysis of multifactorial interventions: (RR 1.29 [0.67, 2.51])^25^ Reduced in 3 studies^77^, ^78^, ^79^ LoS Reduced in 3 studies^58^, ^67^, ^78^ ED visits Reduced in 2 studies^78^, ^79^ Readmissions Reduced in people with more severe dementia^71^, ^76^
	3 (3) 4370	Virtual case management	Hospital admissions No effect^75^, ^80^, ^81^
Critically low	2 (0) 1300	Fam‐FFC	LoS No effect^82^ Readmissions Reduced^82^
		Collaborative dementia care	Hospital admissions; ED visits; readmissions Reduced^78^

*Note*: 95% CIs in square brackets.

Abbreviations: *β*, beta coefficient; CI, confidence interval; DID, difference‐in‐differences estimate per 1000 patients; ED, emergency department; fam‐FFC, family‐focused function‐centered; LoS, length of stay; MD, mean difference; OR, odds ratio; PwD, people with dementia; RR, risk ratio.

#### 3.1.1 High quality

One review of five studies examining the effects of case management interventions reported no significant reduction in hospital admissions.^30^ One study in this review found a significant decrease in the number of days in a hospital unit or residential home in the case management group at 6 and 12 months, although it was not possible to differentiate hospital and residential home stays.^58^ A meta‐analysis of three studies reported longer LoS in those who received case management than the control group.^59^, ^60^, ^61^

#### 3.1.2 Moderate quality

##### 3.1.2.1 Case management

Thirteen studies reported no significant differences between groups for hospital admission,^58^, ^59^, ^60^, ^62^, ^63^, ^64^, ^65^, ^66^, ^67^, ^68^, ^69^, ^70^, ^71^, ^72^ 10 studies found no significant difference between groups for ED visits,^58^, ^65^, ^67^, ^68^, ^69^, ^72^, ^73^ and six studies found no significant difference between groups for LoS.^58^, ^59^, ^63^, ^64^, ^67^, ^69^, ^70^ However, one review included unpublished data from a RCT that reported a significant reduction in hospital admissions and LoS in the case management group compared to controls.^74^ Moreover, one study found greater LoS in the case management group,^72^ while another found a small reduction in LoS for case management group.^68^

##### 3.1.2.2 Virtual case management

One RCT reported no significant effect of case management intervention on hospital admissions at either 6 or 12 months compared to the control group.^75^

#### 3.1.3 Low quality

##### 3.1.3.1 Case management

Eight studies found no significant differences between groups for hospital admissions^60^, ^62^, ^63^, ^64^, ^65^, ^66^, ^69^, ^76^ and a small meta‐analysis found no effect of multifactorial interventions and treatments on hospital admissions^25^ even after making adjustments in sensitivity analyses.

Hospital admissions for ambulatory care‐sensitive conditions decreased in the case management group compared to controls after 6 months.^77^ The Healthy Aging Brain Centre reported reductions in ED visits, hospital admissions, and LoS compared to controls.^78^ Another study also found a reduction in ED visits compared to the control group,^79^ and unpublished data from two additional studies demonstrated weak effects on reduced LoS.^58^, ^67^ Moreover, among people with dementia with at least one hospital admission, those with severe cognitive impairment had fewer hospital admissions after 6 months compared to those with milder cognitive impairment.^71^, ^76^

##### 3.1.3.2 Virtual case management

Three studies reported no significant effect of technology‐enabled case management on hospital admissions at 6‐, 12‐, or 24‐month follow‐ups.^75^, ^80^, ^81^

#### 3.1.4 Critically low quality

##### 3.1.4.1 Family‐focused function‐centered care (fam‐FFC)

One study reported no significant difference in LoS between the fam‐FFC group and controls. However, people with dementia receiving fam‐FFC had a significantly reduced risk of 30‐day readmissions.^82^

##### 3.1.4.2 Collaborative dementia care

One study reported that people with dementia receiving collaborative dementia care had lower rates of readmission, ED visits, and hospital admissions compared to controls.^78^

#### 3.1.5 Summary

There was high‐quality evidence to suggest that case management is not effective in reducing hospital admissions and may increase LoS in people with dementia and moderate‐ and low‐certainty evidence suggesting virtual case management is also ineffective.

### 3.2 Multidisciplinary teams

Included MDT interventions varied in structure and composition, but these were often characterized by multidisciplinary services from various healthcare providers, including, neurologists, geriatricians, social workers, nurse practitioners, and physical/occupational therapists.^55^, ^83^ Results are summarized in Table 3.

**TABLE 3:** Summary of evidence and quality of findings for effect of psychosocial interventions on hospitalization in people with dementia.

Intervention	Quality	No. studies (RCTs), sample size	Intervention subtype	Results
Working with people with dementia and carers				
Physical activity	High	6 (6) 1312	Exercise program	Hospital admissions No effect: main analysis (RR 1.10 [0.85, 1.43]), sensitivity analyses (OR 1.21 [0.80, 1.83])^36^
	Low	5 (5) 791	Physiotherapy and occupational therapy	Hospital admissions No effect: (RR 0.83 [0.30, 2.34])^84^ (RR 1.30 [0.63, 2.65])^85^ LoS No effect: (MD 0.16 [−0.36, 0.03])^53^
			Exercise program	Hospital admissions No effect: Home‐based exercise (IR 0.47 [0.31, 0.68]), group‐based exercise (IR 0.54 [0.46, 0.90]), control groups (IR 0.65 [0.46, 0.90])^86^
Group activities	Low	2 (2) 213	Day care	Hospital admissions No effect: (RR 1.15 [0.70, 1.91])^87^, ^88^
	Critically low	1 (0) 85	Music program	LoS Reduced: (34.68 days after intervention vs 36.90 days before intervention),^89^ discharges increased by 9.84%^89^
Counseling	Low	3 (3) 858	Individual and group counseling	Hospital admissions No effect: (RR 0.94 [0.77, 1.14]) at 3 years^90^ (RR 0.81 [0.41, 1.60]) at 12 months^91^ ED visits No effect: (RR 1.00 [0.78, 1.27]) at 3 years^90^
			Reminiscence therapy	Hospital admissions No effect: (RR 1.17 [0.63, 2.20])^92^
Carer interventions	Low	1 (1) 197	REACH‐II	Hospital admissions No effect: (RR 1.09 [0.77, 1.55]) at 12 months^93^ ED visits No effect: (RR 1.26 [0.95, 1.67]) at 12 months^93^
ACP	Low	6 (4) 479,231	ACP facilitation	Hospital admissions Reduced by 55% over 2 years^94^
			Written AD	Hospital admissions Increased by lack of a written AD: (ARR 1.15 [1.14, 1.17])^95^ LoS Reduced with a written AD: (5.7 days (SD 12.6) versus 12.8 days (SD 20.9); *p* = 0.026)^96^
			Staff ACP training	Hospital admissions Reduced^97^ LoS No effect^98^ Reduced^97^
Working with staff and organizational structures				
MDT	Moderate	2 (2) 858	Pharmacist‐led medication reviews	ED visits No effect^99^ Hospital admissions No effect^100^ Reduced after excluding individuals with heart failure^100^
	Low	5 (3) 1982	Pharmacist in MDT	Readmission Reduced medication‐related 30‐day readmissions: (5% vs 11% in controls, *p* = 0.03)^100^
			MDT during transition to assisted living	Hospital admissions No effect: (RR 0.65 [0.26, 1.65])^101^
			Comprehensive geriatric assessment	Hospital admissions No effect: (RD 0.79 [0.50, 1.27]) at 6 months^102^ ED visits No effect: (RD 1.11 [0.44, 2.78])^102^ Readmission No effect^102^
			Geriatric emergency medicine unit for elderly patients	Hospital admissions Increased: (57.8% vs 47.1% compared to standard ED care)^103^ Readmission Reduced: (OR 0.65 [0.46, 0.94] compared to standard ED care)^103^
			ER2 tool	Hospital admissions Reduced in 39%^104^ LoS Increased in ED: (∼4.28 to 5.56 h, *p* < 0.001)^104^
	Critically low	6 (1) 972,253	Virtual MDT	Hospital admissions Reduced^105^, ^106^, ^107^
			Post‐operative MDT	LoS No effect: (Intervention 20.0 ± 12.0 days vs control 32.1 ± 35.3 days; *p* = 0.059)^108^
			MDT during relocation to assisted living	Hospital admissions No effect (*p* = 0.13)^101^ ED visits No effect (*p* = 0.80)^101^
			Home healthcare from MDT	Readmission No effect: (OR = 1.01 [1.00, 1.02]) in comparison with skilled nursing facility^109^
Education/Training	Low	2 (2) 596	Educational material	Hospital admissions No effect: (RR = 0.91 [0.74, 1.13])^110^
			Medication review education	Hospital admissions No effect^111^, ^112^
	Critically low	3 (1) 2033	Dementia training for staff	Hospital admissions No effect at 6 months^113^ LoS No effect but downward trend at end of intervention^114^ Reduced by 6 days (*p* = 0.019)^115^
Type of hospital setting	Moderate	2 (1) 1500	Specialist inpatient dementia units	Hospital admissions Reduced (22% in SIDU vs 28.3% in SWC; *p* = 0.25)^116^ LoS No effect: (SIDU 28.5 days (SD 31.4) versus SWC 25.1 days (SD 48.7); *p* = 0.47)^116^ (SIDU 16 median days vs. SWC 16 median days; *p* = 0.32)^117^ Readmission No effect: (32% in SIDU vs 35% in SWC)^117^
	Critically low	3 (0) 248	Single versus multi‐bed wards	LoS Increased: 62.2 days in single rooms versus 42.5 in multi‐bed wards *p* = 0.027,^118^ 39.7 days in single rooms versus 21.8 in multi‐bed wards, *p* = 0.001^119^ Readmission No effect after 30 days in single rooms (*p* = 0.335)^118^
			Cognitive geriatric unit	LoS Increased: (19.9 ± 4.9 days vs 17.7 ± 4.7 days on control ward)^120^
Discharge intervention	Critically low	2 (0) 433	Post‐discharge follow‐up	LoS Reduced (25 days vs 31 days in control group; *p* = 0.005)^121^ Readmission No effect after 30 days: (8.4% vs 8.0% before intervention; *p* = 0.82)^122^
Palliative care	Moderate	1 (1) 64	Nursing home‐based palliative care	Hospital admissions No effect (OR = 1.06 [0.45, 2.50])^123^ ED visits No effect (OR = 0.89 [0.35, 2.25])^123^
	Critically low	1 (0) 368	Home‐based palliative care	Readmission Reduced (11% vs 35% in matched controls; *p* = 0.01)^124^
Volunteer	Critically low	2 (0) 564	Person‐centered volunteers	LoS Increased (*z* = 3.22; *p* = 0.001; *d* = 0.30)^89^ Readmission No discharged patients readmitted within 28 days^125^

*Note*: 95% confidence intervals presented are in square brackets.

Abbreviations: ACP, advance care planning; AD, advance directives; ARR, adjusted risk ratio; ED, emergency department; ER2 tool, Emergency Room Evaluation and Recommendations Tool; ICU, intensive care unit; IR, incidence rate; LoS, length of stay; MD, mean difference; MDT, multidisciplinary team; OR, odds ratio; RD, risk difference; REACH‐II, Resources for Enhancing Alzheimer's Caregiver Health; RR, risk ratio; SD, standard deviation; SIDU, specialist inpatient dementia units; SWC, standard ward care.

#### 3.2.1 Moderate quality

Two RCTs (*n* = 858) found no effect of pharmacist‐led medication reviews on hospital admissions or ED visits,^99^, ^100^ but one study found a significant decrease in hospitalization after adjusting for heart failure.^100^

#### 3.2.2 Low quality

One RCT (*n* = 460) found that including a clinical pharmacist in the hospital MDT produced significantly lower medication‐related 30‐day readmissions compared to control.^100^ However, comprehensive geriatric assessments and MDT assistance during transition to assisted living did not affect hospitalization.^101^, ^102^

#### 3.2.3 Critically low quality

Video consultations with MDT showed significant evidence of reduced hospital admissions.^105^, ^106^, ^107^ However, post‐operative MDT interventions, MDT during relocation to assisted living, and home healthcare groups found no significant reductions in hospitalization.^101^, ^108^, ^109^

#### 3.2.4 Summary

Moderate‐quality evidence suggests pharmacist‐led medication reviews can reduce hospitalization in dementia only after adjusting for physical comorbidities.

### 3.3 Physical activity

Physical activity interventions included an intervention focused on improving strength, mobility, and balance through exercise groups or physical therapy programs. Results are summarized in Table 3.

#### 3.3.1 High quality

One review of six RCTs examining the effects of supervised exercise programs at home found no significant impact on hospitalizations compared to controls, even after conducting sensitivity analysis.^126^

#### 3.3.2 Low quality

One RCT (*n* = 210) also included in the high‐quality review found hospitalization did not differ across home‐based exercise, group‐based exercise, or control groups.^86^ A further RCT (*n* = 236) found there were also no significant reductions in hospital admissions after physiotherapy and occupational therapy interventions, but there was weak evidence that physiotherapy and occupational therapy were associated with small reductions in LoS.^85^

#### 3.3.3 Summary

There is high‐quality evidence that, while in general supervised exercise programs for people with dementia may be helpful in other domains, they do not decrease the likelihood of hospitalizations.

### 3.4 Education/training

Interventions were categorized as education/training programs provided they aimed to improve staff confidence, knowledge or understanding of dementia care through face‐to‐face training, collaborative meetings, or reading materials. Results are summarized in Table 3.

#### 3.4.1 Low quality

Three RCTs (*n* = 596) assessing educational materials, meetings with healthcare professionals produced, and medication review education found no significant association with hospital admissions compared to controls.^110^, ^111^, ^112^

#### 3.4.2 Critically low quality

While one NRSI (*n* = 68) reported dementia training for staff produced a significant reduction in LoS,^115^ another NRSI (*n* = 661) reported no significant difference in LoS, but a downward trend was established toward the end of the intervention.^114^ One RCT (*n* = 1304) found no effect for nursing home staff training on hospital admissions at 6 months.^113^

#### 3.4.3 Summary

There is no good evidence of an effect of education or training on hospitalization; however, the evidence base is limited.

### 3.5 Type of hospital setting

Type of hospital setting interventions focused on situational variables in acute or community care that differed from regular care settings or were tailored to people with dementia. Results are summarized in Table 3.

#### 3.5.1 Moderate quality

Two studies (*n* = 1500) found no significant difference reported between specialist inpatient dementia units and control groups for LoS.^116^, ^117^ The RCT (*n* = 600) found non‐significant reductions in readmission rates,^117^ and a NRSI (*n* = 900) reported fewer short‐term admissions in the intervention group compared to controls.^116^

#### 3.5.2 Critically low quality

Two NRSIs (*n* = 200) examining single‐bed rooms were associated with significantly increased LoS compared to multi‐bed wards,^118^, ^119^ but there was no significant difference in 30‐day readmissions.^118^ Cognitive Geriatric Units (NRSI, *n* = 48) were also associated with increased LoS compared to control.^120^

#### 3.5.3 Summary

There was a lack of good‐quality evidence, but to date there is no evidence that the type of hospital setting affects hospitalization.

### 3.6 Group activities

Group activities referred to social activities based within the community or residential care settings. Results are summarized in Table 3.

#### 3.6.1 Low quality

Two RCTs (*n* = 213) found day care interventions had no effect on hospitalization compared to control.^87^, ^88^

#### 3.6.2 Critically low quality

One NRSI (*n* = 85) found music programs demonstrated a small reduction in LoS and an increase in the total number of discharges compared to the control group.^89^

#### 3.6.3 Summary

There is no good evidence of an effect of group activities on hospitalization; however, the evidence base is limited.

### 3.7 Discharge intervention

Discharge interventions consisted of follow‐ups from healthcare providers, guidance for people with dementia and caregivers, medication management, or discharge planning for people with dementia discharged from acute care. Results are summarized in Table 3.

#### 3.7.1 Critically low quality

One NRSI (*n* = 390) found no significant differences between post‐discharge and control groups for 30‐day ED readmissions.^122^ Another NRSI (*n* = 43) reported evidence of a significant reduction in LoS.^121^

#### 3.7.2 Summary

There is no good evidence of an effect of discharge intervention on hospitalization; however, the evidence base is limited.

### 3.8 Palliative care

Based on previous definitions, palliative care interventions were categorized as interdisciplinary patient‐centered care focusing on physical, psychosocial, and spiritual needs with careful reviews and management to best promote quality of life.^127^, ^128^ Results are summarized in Table 3.

#### 3.8.1 Moderate quality

One RCT (*n* = 64) reported no significant association between palliative care and ED visits or hospital admissions compared to the control group.^123^

#### 3.8.2 Critically low quality

One observational study (*n* = 368) reported that people with dementia receiving home‐based palliative care had a significantly lower risk of readmissions compared to matched controls.^124^

#### 3.8.3 Summary

There is no good‐quality evidence that palliative care reduces hospital admissions or ED visits on hospitalization; however, the evidence base is limited.

### 3.9 Counseling

Counseling interventions included individual or group‐based talking sessions led and managed by healthcare providers. Results are summarized in Table 3.

#### 3.9.1 Low quality

Three RCTs (*n* = 858) found no significant effect for individual/group counseling or reminiscence therapy on hospitalization.^90^, ^91^, ^92^

#### 3.9.2 Summary

There is no good evidence for an effect of counseling on hospitalization; however, the evidence base is limited.

### 3.10 Volunteers

Volunteer programs were categorized as interventions focusing on social connections and emotional support between volunteers and PwD or caregivers through activities or discussions to enhance quality of life. Results are summarized in Table 3.

#### 3.10.1 Critically low

All patients (*n* = 16) discharged following a meaningful engagement intervention remained out of the hospital for over 28 days.^89^ Also, the person‐centered volunteer intervention had a significantly longer LoS than the control group.^125^

#### 3.10.2 Summary

There is no good evidence of an effect of volunteers on hospitalization; however, the evidence base is limited.

### 3.11 Carer intervention

Carer interventions refer to individualized psychosocial support for caregivers of people with dementia including but not limited to social support, stress management, or skills training. Results are summarized in Table 3.

#### 3.11.1 Low quality

One RCT (*n* = 197) found carer interventions did not have a significant effect on ED visits or hospital admissions after 12 months.^93^

#### 3.11.2 Summary

There is no good evidence of an effect of carer interventions on hospitalization; however, the evidence base is limited.

### 3.12 Advance care plans (ACP)

Advance care planning aims to offer the opportunity to have meaningful discussions planning future care and support, including medical treatment and end‐of‐life support, while people have the mental capacity to do so.^129^ Results are summarized in Table 3.

#### 3.12.1 Low quality

In one review of six studies (four RCTs) and 479,231 participants, ACP facilitation was found to reduce hospital admissions by 55% over 2 years^94^ and written directives found to reduce LoS.^96^, ^130^ Also, the absence of an advance directive was found to increase the risk of hospital admission.^95^ However, training programs for delivering ACP found mixed effects on the LoS compared to control.^97^

#### 3.12.2 Summary

There was only low‐quality evidence of ACP and written directives, but results were promising, suggesting they reduced hospital admission. Training staff to increase delivery of ACP had mixed results.

## 4 DISCUSSION

Our umbrella review is the first to examine the effects of psychosocial and healthcare interventions on reducing hospitalization in PwD. We found 25 systematic reviews, including 77 unique studies (RCTs = 47; NRSIs = 30) with 1,483,077 participants. Overall, there was high certainty that case management and exercise programs had no significant effect on hospital admissions or LoS. The most promising interventions were ACP facilitation, which resulted in significantly reduced hospitalizations and including clinical pharmacists in MDT interventions, which significantly reduced medication‐related readmissions, but both had low/moderate certainty of evidence for efficacy. There was moderate‐certainty evidence that palliative care did not reduce hospitalizations. Other interventions, including education, group activities, counseling, and carer interventions, were not shown to affect hospitalization, but there was low certainty for this evidence.

We conducted a comprehensive search and employed a rigorous systematic approach to data collection and synthesis. Nonetheless, there are some important limitations. First, most evidence was from high‐income countries, and we cannot generalize our findings to low‐ and middle‐income countries or healthcare systems where the availability of services and cultural considerations about care at home are likely to differ significantly. Second, the heterogeneity across interventions and outcomes prevented us from meta‐analyzing findings and categorizing results definitively by intervention and outcome. Third, by definition, umbrella reviews omit recent primary studies of potential value, and therefore our review cannot include the full scope of primary research despite updated searches. Fourth, our use of a single reviewer to assign GRADE ratings to included studies has the potential for error, though the structured GRADE rating checklist we used and team discussions mitigated this. Fifth, while we considered confounders during GRADE assessments, we were unable to establish the influence of confounders on the association between interventions and hospitalization in PwD in non‐randomized studies of interventions. Sixth, several reviews included unpublished data obtained through personal correspondence, and despite contacting authors for clarification, we cannot validate these data and results. Seventh, little research evidence was of high certainty, meaning we cannot establish the true effect of each intervention on hospitalization in dementia without further robust and high‐quality RCTs. Finally, there is a lack of data on cost and cost‐effectiveness, meaning it is not possible to draw conclusions about the economic implications of interventions aimed at reducing hospitalizations.

The most studied intervention was case management, reflecting the expectation that the presence of a clinical lead (case manager) responsible for advocating for, planning, and facilitating access to healthcare services to support the individual and family would be effective in reducing hospitalization. However, we did not find this; instead, there was high‐quality evidence of a small increase in LoS in those receiving case management, possibly due to case managers facilitating healthcare services to support the individual and family members and leading to greater health service use.

Our findings of reduced hospitalization following ACP facilitation and written directives are consistent with a previous review.^131^ Training programs were not effective, possibly because of varied delivery settings or funding, as staff turnover, staff shortages, and organizational culture play a critical role in the successful implementation of ACP.^132^

Previous systematic reviews assessing exercise effects on hospitalization in older adults found a reduction in the number of falls following exercise interventions.^36^, ^133^ However, in our analysis of studies of PwD, we found strong evidence that these programs did not reduce hospitalization, suggesting that there may be additional components required beyond general exercise programs to reduce hospitalization in PwD. Studies of general older adult populations had similarly found that palliative care had a significant effect on hospitalization,^134^, ^135^ but we found with moderate certainty that there was no effect in PwD. This suggests that palliative care needs to be specifically tailored for PwD, such as using specific protocols for observing pain and distress and facilitating decisions with carers to initiate palliative treatment, particularly where people with dementia lack mental capacity.^136^

The low certainty of evidence examining the effects of MDTs on hospitalization in dementia is related to small studies lacking sufficient statistical power. However, one review stated that included primary studies only measured “acute hospital use,” meaning potentially avoidable admissions might have been excluded,^45^ which may explain the absence of an observed association. Previous research on the impact of specialist wards on hospitalization in dementia indicated a potential reduction in readmission^137^ and non‐significant reductions in LoS.^138^ Our results are in line with this, but there is insufficient evidence for firm conclusions.

As the evidence assessing the remaining psychosocial interventions is of critically low quality, we are unable to make any recommendations based on the literature. Consequently, further high‐quality RCTs implementing standardized interventions and outcomes is required to determine their efficacy in reducing hospitalization in PwD.

### 4.1 Conclusion and recommendations

This umbrella review has shown that current evidence for psychosocial and healthcare interventions in reducing hospitalizations in dementia is insufficient to formulate definitive guidelines for commissioners and policymakers. Clinicians may, however, consider ACP or written directives as effective interventions for possibly reducing avoidable admissions, although this evidence is currently of low certainty. Including pharmacists in MDT interventions may also help reduce hospitalizations in dementia care. However, clinicians should acknowledge the high‐certainty evidence suggesting case management and exercise programs are ineffective in reducing hospitalizations in PwD. Robust RCTs will be required to improve the certainty of positive recommendations. Specifically, researchers should focus on interventions that show promise but lack sufficient high‐quality evidence to support them, such as ACP, MDT interventions, and discharge interventions. Moreover, detailed descriptions of interventions and outcomes using standardized checklists, such as the template for intervention description and replication (TIDieR), are needed to reduce heterogeneity and determine the true efficacy of each intervention component. Researchers should also explore the efficacy of these interventions across a broad range of countries and their healthcare systems to establish effectiveness in more diverse settings.

## CONFLICT OF INTEREST STATEMENT

G.L. has received grants from University College London (UCL) Hospitals National Institute for Health and Care Research (NIHR) Biomedical Research Centre, NIHR, North Thames NIHR Applied Research Collaboration, the Alzheimer's Association and Brain Canada, the Norwegian Research Council, Wellcome Trust, payment for presentations by Fondazione Prada, and travel support for attending meetings from global education and career consultants. N.M. has received grants from NIHR, NIHR Three Schools funding, and Alzheimer's Research UK and consulted for Glaxo SmithKline on virus‐related dementia risk. C.R. is supported by the Makaton Charity. K.W. has received grants from NIHR and the Alzheimer's Society. A.S. has received grants from The Geller Commission, the Wellcome Trust, the Alzheimer's Association, and Brain Canada and NIHR and an honorarium for a presentation from Fondazione Prada. C.H., N.A., D.D., A.F.‐S., C.G., S.G., and M.H. have nothing to declare. Author disclosures are available in the Supporting Information.

## Supporting information



Supporting Information

Supporting Information
