# Supplementary material for: The effect of psychosocial and healthcare interventions on reducing hospitalization in people with dementia: an umbrella review
Source: Alzheimers Dement. 2026 Jul 20;22(7):e71323. doi: 10.1002/alz.71323 (PMC13383009; doi:10.1002/alz.71323)
Supplement: Supplementary file 1 — Supporting Information [file ALZ-22-e71323-s001.docx]

**Appendix**

Contents

[**Appendix table 1: PRIOR checklist** 2](#_Toc221275041)

[**Appendix table 2. Search strategy** 6](#_Toc221275042)

[**Appendix table 3. Reasons for study exclusion at full-text review** 7](#_Toc221275043)

[**Appendix table 4: Quality assessment with AMSTAR II tool** 11](#_Toc221275044)

[**Appendix table 5: Grade ratings** 13](#_Toc221275045)

[**Appendix figure 1. Citation Matrix** 15](#_Toc221275046)

# **Appendix table 1: PRIOR checklist**

| Section Topic | # | Item | Location reported |
| --- | --- | --- | --- |
| TITLE | |  |  |
| Title | 1 | Identify the report as an overview of reviews. | Pg 1 |
| ABSTRACT | |  |  |
| Abstract | 2 | Provide a comprehensive and accurate summary of the purpose, methods, and results of the overview of reviews. | Pg 2 |
| INTRODUCTION | |  |  |
| Rationale | 3 | Describe the rationale for conducting the overview of reviews in the context of existing knowledge. | Pg 3 |
| Objectives | 4 | Provide an explicit statement of the objective(s) or question(s) addressed by the overview of reviews. | Pg 3 |
| METHODS | |  |  |
| Eligibility criteria | 5a | Specify the inclusion and exclusion criteria for the overview of reviews. If supplemental primary studies were included, this should be stated, with a rationale. | Pg 4 |
|  | 5b | Specify the definition of ‘systematic review’ as used in the inclusion criteria for the overview of reviews. | Pg 4 |
| Information sources | 6 | Specify all databases, registers, websites, organizations, reference lists, and other sources searched or consulted to identify systematic reviews and supplemental primary studies (if included).  Specify the date when each source was last searched or consulted. | Pg 3 |
| Search strategy | 7 | Present the full search strategies for all databases, registers and websites, such that they could be reproduced. Describe any search filters and limits applied. | Pg 3 |
| Selection process | 8a | Describe the methods used to decide whether a systematic review or supplemental primary study (if included) met the inclusion criteria of the overview of reviews. | Pg 4 |
|  | 8b | Describe how overlap in the populations, interventions, comparators, and/or outcomes of systematic reviews was identified and managed during study selection. | Pg 4 |
| Data collection process | 9a | Describe the methods used to collect data from reports. | Pg 4 |
|  | 9b | If applicable, describe the methods used to identify and manage primary study overlap at the level of the comparison and outcome during data collection. For each outcome, specify the method used to illustrate and/or quantify the degree of primary study overlap across systematic reviews. | Pg 4 |
|  | 9c | If applicable, specify the methods used to manage discrepant data across systematic reviews during data collection. | Pg 4 |
| Data items | 10 | List and define all variables and outcomes for which data were sought. Describe any assumptions made and/or measures taken to identify and clarify missing or unclear information. | Pg 4 |
| Risk of bias assessment | 11a | Describe the methods used to *assess* risk of bias or methodological quality of the included systematic reviews. | Pg 5 |
|  | 11b | Describe the methods used to *collect* data on (from the systematic reviews) and/or *assess* the risk of bias of the primary studies included in the systematic reviews. Provide a justification for instances where flawed, incomplete, or missing assessments are identified but not re-assessed. | Pg 5 |
|  | 11c | Describe the methods used to *assess* the risk of bias of supplemental primary studies (if included). |  |
| Synthesis methods | 12a | Describe the methods used to summarize or synthesize results and provide a rationale for the choice(s). | Pg 5 |
|  | 12b | Describe any methods used to explore possible causes of heterogeneity among results. |  |
|  | 12c | Describe any sensitivity analyses conducted to assess the robustness of the synthesized results. |  |
| Reporting bias assessment | 13 | Describe the methods used to *collect* data on (from the systematic reviews) and/or *assess* the risk of bias due to missing results in a summary or synthesis (arising from reporting biases at the levels of the systematic reviews, primary studies, and supplemental primary studies, if included). | Pg 5 |
| Certainty assessment | 14 | Describe the methods used to *collect* data on (from the systematic reviews) and/or *assess* certainty (or confidence) in the body of evidence for an outcome. | Pg 5 |
| RESULTS | |  |  |
| Systematic review and supplemental primary study selection | 15a | Describe the results of the search and selection process, including the number of records screened, assessed for eligibility, and included in the overview of reviews, ideally with a flow diagram. | Pg 5 |
|  | 15b | Provide a list of studies that might appear to meet the inclusion criteria, but were excluded, with the main reason for exclusion. | Appendix |

| Characteristics of systematic reviews and supplemental primary studies | 16 | Cite each included systematic review and supplemental primary study (if included) and present its characteristics. | Pg 6 |
| --- | --- | --- | --- |
| Primary study overlap | 17 | Describe the extent of primary study overlap across the included systematic reviews. | Pg 6 |
| Risk of bias in systematic reviews, primary studies, and supplemental primary studies | 18a | Present assessments of risk of bias or methodological quality for each included systematic review. | Pg 14 |
|  | 18b | Present assessments (*collected* from systematic reviews or *assessed* anew) of the risk of bias of the primary studies included in the systematic reviews. | Pg 14 |
|  | 18c | Present assessments of the risk of bias of supplemental primary studies (if included). |  |
| Summary or synthesis of results | 19a | For all outcomes, summarize the evidence from the systematic reviews and supplemental primary studies (if included). If meta-analyses were done, present for each the summary estimate and its precision and measures of statistical heterogeneity. If comparing groups, describe the direction of the effect. | Pg 14-26 |
|  | 19b | If meta-analyses were done, present results of all investigations of possible causes of heterogeneity. |  |
|  | 19c | If meta-analyses were done, present results of all sensitivity analyses conducted to assess the robustness of synthesized results. |  |
| Reporting biases | 20 | Present assessments (*collected* from systematic reviews and/or *assessed* anew) of the risk of bias due to missing primary studies, analyses, or results in a summary or synthesis (arising from reporting biases at the levels of the systematic reviews, primary studies, and supplemental primary studies, if included) for each summary or synthesis assessed. | Pg 14 |
| Certainty of evidence | 21 | Present assessments (*collected* or *assessed* anew) of certainty (or confidence) in the body of evidence for each outcome. | Pg 14 |
| DISCUSSION | | |  |
| Discussion | 22a | Summarize the main findings, including any discrepancies in findings across the included systematic reviews and supplemental primary studies (if included). | Pg 27 |
|  | 22b | Provide a general interpretation of the results in the context of other evidence. | Pg 27/28 |
|  | 22c | Discuss any limitations of the evidence from systematic reviews, their primary studies, and supplemental primary studies (if included) included in the overview of reviews. Discuss any limitations of the overview of reviews methods used. | Pg 27 |
|  | 22d | Discuss implications for practice, policy, and future research (both systematic reviews and primary research). Consider the relevance of the findings to the end users of the overview of reviews, e.g., healthcare providers, policymakers, patients, among others. | Pg 28 |
| OTHER INFORMATION | | |  |
| Registration and protocol | 23a | Provide registration information for the overview of reviews, including register name and registration number, or state that the overview of reviews was not registered. | Pg 2/3 |
|  | 23b | Indicate where the overview of reviews protocol can be accessed, or state that a protocol was not prepared. | Pg 2/3 |
|  | 23c | Describe and explain any amendments to information provided at registration or in the protocol. Indicate the stage of the overview of reviews at which amendments were made. | Pg 3 |
| Support | 24 | Describe sources of financial or non-financial support for the overview of reviews, and the role of the funders or sponsors in the overview of reviews. | Pg 30 |
| Competing  interests | 25 | Declare any competing interests of the overview of reviews' authors. | Pg 30 |
| Author information | 26a | Provide contact information for the corresponding author. | Pg 1 |
|  | 26b | Describe the contributions of individual authors and identify the guarantor of the overview of reviews. | Pg 30/21 |
| Availability of data and other materials | 27 | Report which of the following are available, where they can be found, and under which conditions they may be accessed: template data collection forms; data collected from included systematic reviews and supplemental primary studies; analytic code; any other materials used in the overview of reviews. | Pg 30 |

# **Appendix table 2. Search strategy**

**2A - Medline / EMBASE**

| 1. Meta-analysis as Topic/ 2. meta analy$.tw. 3. metanaly$.tw. 4. Meta-Analysis/ 5. (systematic adj (review$1 or overview$s1)).tw. 6. exp Review Literature as Topic/ 7. or/1-6 8. cochrane.ab. 9. embase.ab. 10. (psych lit or psyclit).ab. 11. (psychinfo or psycinfo).ab. 12. (cinahl or cinhal).ab. 13. science citation index.ab. 14. bids.ab. 15. cancerlit.ab. 16. or/8-15 17. reference list$.ab. 18. bibliograph$.ab. 19. hand-search$.ab. 20. relevant journals.ab. 21. manual search$.ab. 22. or/17-21 23. selection criteria.ab. 24. data extraction.ab. 25. 23 or 24 26. Review/ 27. 25 and 26 28. Comment/ 29. Letter/ 30. Editorial/ 31. animal/ 32. human/ 33. 31 not (31 and 32) 34. or/28-30,33 35. 7 or 16 or 22 or 27 | 1. 35 not 34 2. (dementia or dement$ or “Alzheimer’s dementia”).tw. 3. Dementia.mp. 4. 37 or 38 5. advance care plan$.mp. 6. case manag$.mp. 7. psychoeduca$.mp. 8. self-manag$.mp. 9. palliative care.mp. 10. advance direct$.tw. 11. Intervention.mp. 12. therapy.mp. 13. evaluat$.mp. 14. train$.mp. 15. rehabilitation.mp. 16. treatment$.mp. 17. or/40-52 18. hospita$.mp. 19. admis$.mp. 20. admit$.mp. 21. readmi$.mp. 22. length of stay.mp. 23. discharg$.mp. 24. Hospitalization/ 25. Emergency Room Visits/ 26. Or/54-61 27. 36 and 39 and 53 and 62 |
| --- | --- |

**2B. Cochrane central register of controlled trials**

| (MH "Hospitalization+" OR MH "Emergency Room Visits" OR hospita* OR readmi* OR length of stay OR discharg*) AND (MH "Psychosocial Intervention" OR advance care plan* OR case manag* OR psychoeduca* OR self-manag* OR palliative care OR advance direct* OR therapy) AND (MH "Systematic Review" OR (meta-analysis or systematic review)) AND (MH "Dementia+" OR ((dementia or dement$ or "Alzheimer's dementia"))) |
| --- |

**2C. CINAHL**

| (MH "Meta Analysis" OR MH "Systematic Review" OR MH "Literature Review+") AND (MH "Dementia+" OR (dementia or alzheimer's) OR lewy body dementia OR vascular dementia) AND (MH "Psychosocial Intervention" OR advance care plan* OR case manag* OR psycheduca* OR self-manage* OR palliative care OR advance direct* OR therapy OR rehabilitation OR treatment) AND (MH "Hospitalization+" OR MH "Emergency Room Visits" OR admis* OR readmi* OR length of stay OR discharg*) |
| --- |

# **Appendix table 3. Reasons for study exclusion at full-text review**

| Could not extract information for people with dementia | **1** | **Optimisation of medications used in residential aged care facilities: a systematic review and meta-analysis of randomised controlled trials**  Almutairi H.; ; Stafford A.; Etherton-Beer C.; Flicker L.  BMC geriatrics / 2020;20(1):236  DOI:[10.1186/s12877-020-01634-4](https://dx.doi.org/10.1186/s12877-020-01634-4) |
| --- | --- | --- |
|  | **2** | **Improving the appropriateness of prescribing in older patients: A systematic review and meta-analysis of pharmacists' interventions in secondary care**  Anthonywalsh K.; ; O'riordan D.; Kearney P.M.; Timmons S.; Byrne S.  Age and Ageing / 2016;45(2):201EP – 209  DOI:[10.1093/ageing/afv190](https://dx.doi.org/10.1093/ageing/afv190) |
|  | **3** | **Integrated Physical‐Mental Healthcare Services in Specialist Settings to Improve Outcomes for Older People Living With Mental Health Diagnoses: A Systematic Review.**  Beishon, Lucy; Hickey, Bethan; Desai, Bhavisha; Chithiramohan, Tamara; Evley, Rachel; Subramaniam, Hari; Maniatopoulos, Gregory; Rajkumar, Anto P.; Dening, Tom; Mukateova‐Ladinska, Elizabeta; Robinson, Thompson G.; Tarrant, Carolyn  International Journal of Geriatric Psychiatry 09// 2024;39(9):1-16  DOI:[10.1002/gps.6146](https://dx.doi.org/10.1002/gps.6146) |
|  | **4** | **Multidisciplinary team interventions for delirium in patients with chronic cognitive impairment**  Britton A.; ; Russell R.  Cochrane database of systematic reviews (Online) / 2000;(2):CD000395 |
|  | **5** | **Impact of home healthcare on end-of-life outcomes for people with dementia: a systematic review**  Chen P.-J.; ; Smits L.; Miranda R.; Liao J.-Y.; Petersen I.; Van den Block L.; Sampson E.L.  BMC geriatrics / 2022;22(1):80  DOI:[10.1186/s12877-022-02768-3](https://dx.doi.org/10.1186/s12877-022-02768-3) |
|  | **6** | **The effectiveness of adult day services for older adults: A review of the literature from 2000 to 2011**  Fields N.L.; ; Anderson K.A.; Dabelko-Schoeny H.  Journal of Applied Gerontology / 2014;33(2):130EP - 163  DOI:[10.1177/0733464812443308](https://dx.doi.org/10.1177/0733464812443308) |
|  | **7** | **Joint geriatric and psychiatric wards: A review of the literature**  George J.; ; Adamson J.; Woodford H.  Age and Ageing / 2011;40(5):543EP - 548  DOI:[10.1093/ageing/afr080](https://dx.doi.org/10.1093/ageing/afr080) |
|  | **8** | **Interventions to Prevent Falls in Older Adults: An Evidence Update for the U.S. Preventive Services Task Force**  Guirguis-Blake, Janelle M.; ; Perdue, Leslie A.; Coppola, Erin L.; Bean, Sarah I.  Agency for Healthcare Research and Quality (US) 2024 |
|  | **9** | **Review article: End‐of‐life care for older people in the emergency department: A scoping review.**  Huang, Ya‐Ling; Alsaba, Nemat; Brookes, Gemma; Crilly, Julia  Emergency Medicine Australasia 02// 2020;32(1):7-19  DOI:[10.1111/1742-6723.13414](https://dx.doi.org/10.1111/1742-6723.13414) |
|  | **10** | **A systematic review to identify and assess the effectiveness of alternatives for people over the age of 65 who are at risk of potentially avoidable hospital admission**  Huntley A.L.; ; Chalder M.; Shaw A.R.G.; Hollingworth W.; Metcalfe C.; Benger J.R.; Purdy S.  BMJ Open / 2017;7(7):016236  DOI:[10.1136/bmjopen-2017-016236](https://dx.doi.org/10.1136/bmjopen-2017-016236) |
|  | **11** | **Long-term home and community-based exercise programs improve function in community-dwelling older people with cognitive impairment: a systematic review**  Lewis M.; ; Peiris C.L.; Shields N.  Journal of physiotherapy / 2017;63(1):23EP - 29  DOI:[10.1016/j.jphys.2016.11.005](https://dx.doi.org/10.1016/j.jphys.2016.11.005) |
|  | **12** | **Psychosocial interventions for reducing antipsychotic medication in care home residents.**  Luhnen, Julia; ; Richter, Tanja; Calo, Stella; Meyer, Gabriele; Kopke, Sascha; Mohler, Ralph  The Cochrane database of systematic reviews / 2023;8(100909747):CD008634  DOI:[10.1002/14651858.CD008634.pub3](https://dx.doi.org/10.1002/14651858.CD008634.pub3) |
|  | **13** | **Volunteer Programs Supporting People With Dementia/Delirium in Hospital: Systematic Review and Meta-Analysis**  Pritchard E.; ; Soh S.-E.; Morello R.; Berkovic D.; Blair A.; Anderson K.; Bateman C.; Moran C.; Tsindos T.; O'Donnell R.; Ayton D.  The Gerontologist / 2021;61(8):e421EP - e434  DOI:[10.1093/geront/gnaa058](https://dx.doi.org/10.1093/geront/gnaa058) |
|  | **14** | **A systematic review of the effectiveness of advance care planning interventions for people with cognitive impairment and dementia.**  Robinson, Louise; Dickinson, Claire; Rousseau, Nicolette; Beyer, Fiona; Clark, Alexa; Hughes, Julian; Howel, Denise; Exley, Catherine  Age & Ageing 03// 2012;41(2):263-269  DOI:[10.1093/ageing/afr148](https://dx.doi.org/10.1093/ageing/afr148) |
|  | **15** | **Association between advanced care planning and emergency department visits: A systematic review.**  Sakamoto, Ayaka; ; Inokuchi, Ryota; Iwagami, Masao; Sun, Yu; Tamiya, Nanako  The American journal of emergency medicine / 2023;68(aa2, 8309942):84-91  DOI:[10.1016/j.ajem.2023.03.004](https://dx.doi.org/10.1016/j.ajem.2023.03.004) |
|  | **16** | **Impact of deprescribing dual-purpose medications on patient-related outcomes for older adults near end-of-life: a systematic review and meta-analysis**  Shrestha S.; ; Poudel A.; Cardona M.; Steadman K.J.; Nissen L.M.  Therapeutic Advances in Drug Safety / 2021;12((Shrestha)  DOI:[10.1177/20420986211052343](https://dx.doi.org/10.1177/20420986211052343) |
|  | **17** | **Populations and Interventions for Palliative and End-of-Life Care: A Systematic Review.**  Singer, Adam E.; Goebel, Joy R.; Kim, Yan S.; Dy, Sydney M.; Ahluwalia, Sangeeta C.; Clifford, Megan; Dzeng, Elizabeth; O'Hanlon, Claire E.; Motala, Aneesa; Walling, Anne M.; Goldberg, Jaime; Meeker, Daniella; Ochotorena, Claudia; Shanman, Roberta; Cui, Mike; Lorenz, Karl A.  Journal of Palliative Medicine 09// 2016;19(9):995-1008  DOI:[10.1089/jpm.2015.0367](https://dx.doi.org/10.1089/jpm.2015.0367) |
|  | **18** | **Enhanced rehabilitation and care models for adults with dementia following hip fracture surgery**  Smith, TO; Gilbert, AW; Sreekanta, A; Sahota, O; Griffin, XL; Cross, JL; Fox, C; Lamb, SE  Cochrane Database of Systematic Reviews 2020;( 2):  DOI:[10.1002/14651858.CD010569.pub3](https://dx.doi.org/10.1002/14651858.CD010569.pub3) |
|  | **19** | **Caregiver-Based Interventions to Optimize Medication Safety in Vulnerable Elderly Adults: A Systematic Evidence-Based Review**  Wagle K.C.; ; Skopelja E.N.; Campbell N.L.  Journal of the American Geriatrics Society / 2018;66(11):2128EP - 2135  DOI:[10.1111/jgs.15556](https://dx.doi.org/10.1111/jgs.15556) |
|  | **20** | **Digital Decision Aids to Support Decision-Making in Palliative and End-of-Life Dementia Care: Systematic Review and Meta-Analysis**  Zhong J.; Liang W.; Wang T.; Chau P.H.; Davies N.; Zhao J.; Chu H.N.C.; Lin C.C.  Journal of Medical Internet Research / 2025;27  DOI:[10.2196/71479](https://dx.doi.org/10.2196/71479) |
| No hospitalization outcome reported | **1** | **Home Hospitalization in Palliative Care for Advanced Cancer and Dementia: A Systematic Review**  Farinha-Costa B.; Reis-Pina P.  Journal of Pain and Symptom Management / 2025;69(3):289EP - 303  DOI:[10.1016/j.jpainsymman.2024.11.013](https://dx.doi.org/10.1016/j.jpainsymman.2024.11.013) |
|  | **2** | **Interventions for improving palliative care for older people living in nursing care homes.**  Hall S; Kolliakou A; Petkova H; Froggatt K; Higginson IJ  Cochrane Database of Systematic Reviews 01// 2011 |
|  | **3** | **A systematic review of the evidence for the value of functional assessment of older people with dementia**  Tullis A.; ; Nicol M.  British Journal of Occupational Therapy / 1999;62(12):554EP - 563  DOI:[10.1177/030802269906201206](https://dx.doi.org/10.1177/030802269906201206) |
| Not a systematic review | **1** | **Social isolation in community-dwelling seniors: an evidence-based analysis.**  Ontario health technology assessment series / 2008;8(5):1-49 |
|  | **2** | **Optimizing the diagnosis and management of dementia within primary care: a systematic review of systematic reviews.**  Fernandes, Brooklynn; Goodarzi, Zahra; Holroyd-Leduc, Jayna  BMC Family Practice 08/11/ 2021;22(1):1-17  DOI:[10.1186/s12875-021-01461-5](https://dx.doi.org/10.1186/s12875-021-01461-5) |
|  | **3** | **The role of the dementia specialist nurse in acute care: a scoping review**  Griffiths P.; ; Bridges J.; Sheldon H.; Thompson R.  Journal of clinical nursing / 2015;24(9-10):1394EP - 1405  DOI:[10.1111/jocn.12717](https://dx.doi.org/10.1111/jocn.12717) |
| Conference abstract | **1** | **The effect of dementia case management in community-dwelling individuals with dementia on resource utilization: A systematic review and meta-analysis**  Cepoiu-Martin M.; ; Tam H.; Maxwell C.; Drummond N.; Ronksley P.; Hemmelrgan B.  Alzheimer's and Dementia / 2012;8(4 SUPPL. 1)  DOI:[10.1016/j.jalz.2012.05.1532](https://dx.doi.org/10.1016/j.jalz.2012.05.1532) |
|  | **2** | **Hospitalization in persons with dementia receiving care coordination**  Lee Y.; ; Amjad H.; Johnston D.; Reuland M.; Willink A.; Davis K.; Lyketsos C.; Samus Q.  Journal of the American Geriatrics Society / 2020;68(SUPPL 1):S174  DOI:[10.1111/jgs.16431](https://dx.doi.org/10.1111/jgs.16431) |

# **Appendix table 4: Quality assessment with AMSTAR II tool**

| **Author** | **Question and inclusion** | **Protocol*** | **Study design** | **Search strategy*** | **Study selection** | **Data extraction** | **Exclusion reasons*** | **Inclusion details** | **RoB assessment (RCTs) *** | **RoB assessment (NRSI) *** | **Funding** | **Meta-analysis method (RCTs)*** | **Meta-analysis method (NRSI)*** | **Meta-analysis RoB impact** | **RoB discussion*** | **Heterogeneity discussion** | **Publication bias*** | **Conflict of interest** | **Overall** |
| --- | --- | --- | --- | --- | --- | --- | --- | --- | --- | --- | --- | --- | --- | --- | --- | --- | --- | --- | --- |
| **Reich** | Yes | Py | Yes | No | Yes | Yes | Yes | Py | No | Py | No | N/A | N/A | N/A | Yes | Yes | N/A | Yes | Low |
| **De Souto Barreto** | Yes | Yes | Yes | Py | Yes | Yes | Yes | Yes | Yes | N/A | No | Yes | N/A | N/A | Yes | Yes | Yes | Yes | High |
| **Pimouguet** | No | No | No | No | No | No | No | Py | No | N/A | No | N//A | N/A | N/A | No | No | N/A | No | Critically low |
| **Dixon** | Yes | No | No | Yes | Yes | No | Py | Py | No | No | No | N/A | N/A | N/A | Yes | No | N/A | Yes | Critically low |
| **Tam-Tham** | Yes | Yes | Yes | Py | Yes | Yes | Yes | Yes | Yes | Py | No | Yes | N/A | Yes | Yes | Yes | Yes | Yes | Moderate |
| **Frost** | Yes | No | Yes | Yes | Yes | Yes | Yes | Py | Py | Py | No | Yes | N/A | Yes | Yes | Yes | No | Yes | Critically low |
| **Phelan** | Yes | No | No | Py | Yes | Yes | Yes | Py | No | N/A | No | N/A | N/A | N/A | Yes | Yes | N/A | Yes | Critically low |
| **Quinn** | Yes | Yes | No | Py | Yes | Yes | Yes | Py | Yes | N/A | No | Yes | N/A | Yes | Yes | Yes | Yes | Yes | Moderate |
| **Lee** | Yes | No | Yes | Yes | Yes | Yes | Yes | Yes | Yes | N/A | No | Yes | N/A | Yes | Yes | Yes | Yes | Yes | Low |
| **Reilly** | Yes | Yes | Yes | Yes | Yes | Yes | Yes | Yes | Yes | N/A | Yes | Yes | N/A | Yes | Yes | Yes | Yes | Yes | High |
| **Ma** | Yes | No | Yes | Py | Yes | Yes | Py | Py | No | No | No | N/A | N/A | N/A | Yes | No | N/A | Yes | Critically low |
| **Sawan** | Yes | No | Yes | Py | Yes | Yes | Yes | Py | N/A | Yes | No | N/A | N/A | N/A | No | Yes | N/A | Yes | Critically low |
| **Demanes** | No | No | No | No | No | No | Py | Yes | Py | N/A | No | N/A | N/A | N/A | No | No | N/A | Yes | Critically low |
| **McCausland** | Yes | Py | Yes | Yes | Yes | Yes | Yes | Py | No | Py | No | N/A | N/A | N/A | Yes | Yes | N/A | Yes | Moderate |
| **Hovsepian** | Yes | No | Yes | Py | Yes | Yes | Yes | Py | Yes | Yes | No | N/A | N/A | N/A | Yes | Yes | N/A | Yes | High |
| **Tunnard** | Yes | Py | Yes | Py | Yes | Yes | Yes | Yes | Py | Py | No | N/A | N/A | N/A | Yes | Yes | N/A | Yes | High |
| **Feast** | Yes | Py | Yes | Yes | No | No | Yes | Yes | Yes | Py | No | N/A | N/A | N/A | Yes | Yes | N/A | Yes | High |
| **Packer** | Yes | No | Yes | Yes | Yes | Yes | Py | Yes | Yes | N/A | No | Yes | N/A | Yes | Yes | Yes | No | Yes | Critically low |
| **Godard-Sebillotte** | Yes | Py | No | Py | Yes | Yes | Yes | Yes | No | N/A | No | Yes | N/A | Yes | Yes | Yes | Yes | Yes | Low |
| **Casarez** | Yes | Py | Yes | Py | Yes | No | Yes | Py | Py | Py | No | N/A | N/A | N/A | Yes | No | N/A | Yes | Moderate |
| **Khanassov** | No | No | Yes | Yes | Yes | Yes | Yes | Yes | Py | Py | No | N/A | N/A | N/A | Yes | No | N/A | Yes | Low |
| **Andrews** | Yes | Yes | Yes | Py | Yes | Yes | Yes | Py | Yes | Yes | No | N/A | N/A | N/A | Yes | Yes | N/A | Yes | Moderate |
| **Bocks** | Yes | Py | Yes | Yes | Yes | Yes | Yes | Yes | No | No | No | N/A | N/A | N/A | No | Yes | N/A | Yes | Critically low |
| **Lee (2025)** | Yes | Yes | Yes | Py | Yes | Yes | Yes | Py | Yes | Py | No | N/A | N/A | N/A | Yes | Yes | N/A | Yes | Moderate |
| **Smith** | Yes | No | Yes | Py | Yes | Yes | Yes | Py | N/A | Yes | No | N/A | N/A | N/A | No | No | N/A | Yes | Critically low |

Key:

Py – partial yes; N/A – no meta-analysis performed

*Critical domain

# **Appendix table 5: Grade ratings**

| **Author (Year)** | **Intervention type** | **No. of studies (of which RCTs)** | **Downgrading** | | | | | **Upgrading** | | | **GRADE quality** |
| --- | --- | --- | --- | --- | --- | --- | --- | --- | --- | --- | --- |
|  |  |  | **Risk of bias** | **Inconsistency** | **Indirectness** | **Imprecision** | **Publication Bias** | **Magnitude of effect** | **Dose-response gradient** | **Direction of bias** |  |
| **Reich (2022)** | Situational | 2 (0) | ⊝ | ⊕ | ⊕ | ⊝ | N/A | Yes | N/A | No | Critically low |
|  | Group activities | 1 (0) | ⊝ | ⊕ | ⊕ | ⊕ | N/A | No | N/A | No |  |
|  | Volunteer | 1 (0) | ⊝ | ⊕ | ⊕ | ⊝ | N/A | No | N/A | No |  |
| **DeSouto Barreto (2021)** | Physical activity | 6 (6) | ⊕ | ⊕ | ⊕ | ⊕ | ⊕ | No | N/A | No | High |
| **Pimouguet (2010)** | Case management | 3 (3) | ⊝ | ⊕ | ⊕ | ⊝ | N/A | No | N/A | No | Low |
| **Dixon (2018)** | ACP | 6 (4) | ⊝ | ⊕ | ⊕ | ⊕ | ⊝ | No | N/A | No | Low |
| **Tam-Tham (2013)** | Case management | 3 (3) | ⊕ | ⊕ | ⊕ | ⊝ | ⊕ | No | N/A | No | Moderate |
| **Frost (2020)** | Case management | 3 (1) | ⊝ | ⊕ | ⊕ | ⊕ | N/A | No | N/A | No | Moderate |
| **Phelan (2015)** | Physical activity | 1 (1) | ⊝ | ⊕ | ⊕ | ⊝ | N/A | No | N/A | No | Low |
|  | MDT | 1 (0) | ⊝ | ⊕ | ⊕ | ⊝ | N/A | No | N/A | No | Critically low |
|  | Case management | 8 (8) | ⊝ | ⊝ | ⊕ | ⊕ | N/A | No | N/A | No | Low |
| **Quinn (2020)** | Case management | 1 (1) | ⊕ | ⊕ | ⊕ | ⊝ | ⊕ | No | N/A | No | Moderate |
|  | Palliative care | 1 (1) | ⊕ | ⊕ | ⊕ | ⊝ | ⊕ | No | N/A | No |  |
| **Lee (2020)** | Physical activity | 1 (1) | ⊝ | ⊕ | ⊕ | ⊝ | N/A | No | N/A | No | Low |
|  | Case management | 8 (7) | ⊝ | ⊝ | ⊕ | ⊕ | N.A | No | N/A | No |  |
| **Reilly (2015)** | Case management | 5 (5) | ⊕ | ⊕ | ⊕ | ⊕ | ⊕ | No | N/A | No | High |
| **Ma (2019)** | Case management | 2 (0) | ⊝ | ⊕ | ⊕ | ⊕ | N/A | No | N/A | No | Critically low |
|  | Palliative care | 1 (0) | ⊝ | ⊕ | ⊕ | ⊝ | N/A | No | N/A | No |  |
|  | Discharge | 1 (0) | ⊝ | ⊕ | ⊕ | ⊝ | N/A | No | N/A | No |  |
|  | MDT | 1 (1) | ⊝ | ⊕ | ⊕ | ⊕ | N/A | No | N/A | No | Low |
| **Sawan (2021)** | Case management | 1 (0) | ⊝ | ⊕ | ⊕ | ⊕ | N/A | No | N/A | No | Critically low |
|  | Discharge | 2 (0) | ⊝ | ⊕ | ⊕ | ⊝ | N/A | No | N/A | No |  |
| **Demanes (2021)** | Case management | 3 (3) | ⊕ | ⊕ | ⊝ | ⊝ | N/A | No | N/A | No | Low |
| **McCausland (2019)** | Type of hospital setting | 2 (1) | ⊕ | ⊕ | ⊕ | ⊕ | N/A | No | N/A | No | Moderate |
| **Hovsepian (2022)** | Case management` | 12 (9) | ⊕ | ⊝ | ⊝ | ⊕ | N/A | No | N/A | No | Low |
| **Tunnard (2022)** | MDT | 3 (1) | ⊕ | ⊕ | ⊕ | ⊝ | ⊕ | No | N/A | No | Critically low |
| **Feast (2020)** | Type of hospital setting | 1 (0) | ⊕ | ⊕ | ⊕ | ⊝ | N/A | No | N/A | No | Critically low |
|  | MDT | 1 (0) | ⊕ | ⊕ | ⊕ |  | N/A | No | N/A | No |  |
|  | Group activities | 1 (0) | ⊕ | ⊕ | ⊕ |  | N/A | No | N/A | No |  |
|  | Volunteer | 1 (0) | ⊕ | ⊕ | ⊕ |  | N/A | No | N/A | No |  |
| **Packer (2019)** | Case management | 8 (8) | ⊝ | ⊕ | ⊕ | ⊕ | N/A | No | N/A | No | Moderate |
|  | Physical activity | 3 (3) | ⊝ | ⊕ | ⊕ | ⊝ | N/A | No | N/A | No | Low |
|  | MDT | 1 (1) | ⊝ | ⊕ | ⊕ | ⊝ | N/A | No | N/A | No |  |
|  | Counselling | 2 (2) | ⊝ | ⊕ | ⊕ | ⊝ | N/A | No | N/A | No |  |
|  | Group activities | 2 (2) | ⊝ | ⊕ | ⊕ | ⊝ | N/A | No | N/A | No |  |
| **Godard-Sebillotte (2019)** | Case management | 11 (11) | ⊝ | ⊕ | ⊕ | ⊕ | ⊕ | No | No | No | Moderate |
|  | Education/ training | 1 (1) | ⊝ | ⊕ | ⊕ | ⊝ | ⊕ | No | No | No | Low |
|  | MDT | 1 (1) | ⊝ | ⊕ | ⊕ | ⊝ | ⊕ | No | No | No |  |
|  | Carer intervention | 1 (1) | ⊝ | ⊕ | ⊕ | ⊝ | ⊕ | No | No | No |  |
|  | Counselling | 2 (2) | ⊝ | ⊕ | ⊕ | ⊝ | ⊕ | No | No | No |  |
| **Casarez (2024)** | Case management | 1 (0) | ⊕ | ⊕ | ⊕ | ⊝ | N/A | No | No | No | Critically low |
|  |  | 2 (0) | ⊕ | ⊕ | ⊕ | ⊝ | N/A | No | No | No |  |
| **Khanassov (2014)** | Case management | 2 (2) | ⊝ | ⊕ | ⊕ | ⊝ | N/A | No | No | No | Low |
| **Andrews (2025)** | Education/ training  MDT | 2 (0)  2 (2) | ⊝  ⊕ | ⊕  ⊕ | ⊕  ⊕ | ⊕  ⊝ | N/A  N/A | No  No | No  No | No  No | Critically low |
|  |  |  |  |  |  |  |  |  |  |  | Moderate |
| **Bocks (2025)** | Education/ training | 1 (1) | ⊝ | ⊕ | ⊕ | ⊕ | N/A | No | No | No | Low |
| **Lee (2025)** | MDT | 2 (0) | ⊕ | ⊕ | ⊕ | ⊕ | N/A | No | No | No | Low |
| **Smith (2025)** | MDT | 1 (0) | ⊝ | ⊕ | ⊕ | ⊕ | N/A | No | No | No | Critically low |

⊕ No concerns

⊝Serious concerns

# **Appendix figure 1. Citation Matrix**
